# Terrain uplift due to natural hydrologic overpressure in karstic conduits

**DOI:** 10.1038/s41598-019-38814-1

**Published:** 2019-03-08

**Authors:** Carla Braitenberg, Tommaso Pivetta, Dora Francesca Barbolla, Franci Gabrovšek, Roberto Devoti, Ildikó Nagy

**Affiliations:** 1 Department of Mathematics and Geosciences, University of Trieste, Trieste, Italy; 2 ZRC SAZU, Karst Research Institute, Postojna, Slovenia; 3 Osservatorio Nazionale Terremoti, Istituto Nazionale di Geofisica e Vulcanologia, Roma, Italy

## Abstract

Water supply from karst sources is a worldwide natural resource and the exploitation is tied to the knowledge of the positions of the hydrologic channels. We show that surface deformation induced by flood events in karst conduits is observable, and consists in uplift and outward movement from the hydraulic channel. Precipitation events produce the natural occurrence of subsurface hydraulic overpressure up to 1 MPa. Numerical modeling shows that the stresses are so strong to uplift and dislocate the surface by several mm and induce tilts in the order of microradians. The naturally induced deformation is compatible with a transient internal pressure loading of a channel. The results can be used to find new channels with dense GNSS networks. Sea water incursion and channels accessed for tourism could be monitored. Seismicity has been shown to have a seasonal variation in some areas, which could be explained by the subsurface stresses induced by the natural subsurface overpressure. The pressure induced deformation is expected to be observed in all karstic systems worldwide.

## Introduction

The goal of our study is to detect underground hydrologic channels through geodetic observations. In karstic formations the channels produce water supply, and are tapped for water provisioning and must be protected against pollution.

The observations we discuss relate to deformation in areas where there is fractured crust and fast flowing underground water, as is typical for all karstic formations, and in particular in the prototypical Karst straddling the Italian-Slovenian border (*Kras/Carso Plateau*). Our findings were triggered by the recent hydrologic models of the pressures building up during floods in the Karst system. This started off numerical finite element modeling of the deformation induced by the pressure buildup. The models are verified by a database of over 50 years of long-base tilt measurements, and the time series of the water flow entering the Karst system over the same period. Furthermore, a decade long GNSS time series is available. In the Karst several large flood events have been observed due to the long observation time histories. The observed signals are well above the instrumental noise level, except for the GNSS where they are at the noise level.

Seasonal surface movements have been observed worldwide with GNSS and described with an annual and semiannual periodicity, on top of which shorter period and irregular transient movements are observed^1^. These movements, with a strong vertical component, have been modeled by elastic response to seasonal surface water storage^2^. Local subsidence in US peaks up to 12 mm in fall due to snow and rain load, and the symmetric uplift is explained by deloading due to weight loss of snow melt, rain run-off, and soil moisture evaporation. In the US the inferred seasonal water change is up to 0.6 m^2^. Uplift of up to 15 mm observed with GNSS since the draught in 2013 in California^3^ and western US^4^ has been shown to correlate to flexural unloading, the water storage loss being the source of unloading. Between 2012 and 2014 the unloading has been attributed^4^ to 50 cm of equivalent water height loss in western US. Fluid extraction has shown to lead to strain and pore pressure signals, in California amounting to up to 0.15 MPa of pore pressure variations in the sediments^5^. Slow land uplift in California between 1992 and 2000 in the Santa Clara Valley and observed by interferometric synthetic aperture radar (InSAR) had been proposed to be connected to pore pressure increase accompanying groundwater redistribution^6^. The relevance of pressure changes is of concern in the Woodville karst plain, Florida, where the sea incursion into the karst hydrologic system due to pressure variations is a challenge for the water supply^7^.

The uplift we describe is due to a natural pressurization of the underground flow channels. Illustrating a selection of flood events, we characterize the pressure induced deformation in terms of amplitude, movement direction and time evolution. We find that the underground flood induces a soil uplift, tilting and lateral movement away from the over- pressured channel. In order to quantify the expected deformation, we use the Finite Element method. The observations can be explained by an elastic half space model with a horizontal channel several km long on which a transient pressure pulse is applied.

## Results

### Karst system and aquifer

Karst aquifers are characterized by networks of solution conduits. Their evolution is guided by the geological structure, lithology, geochemical and hydraulic boundary conditions, which are unique for each particular site. These conditions give rise to different processes and mechanisms, which in turn, drive the system towards *equilibrium* when all the recharge is efficiently drained to the springs along the most efficient pathways^8–10^. In tectonically active areas, karst systems continuously adapt to relatively rapid changes of structural and boundary conditions^11,12^. Most of these systems are far from equilibrium; the flow pathways exhibit high variability of channel cross-sections and breakdowns, which restrict the flow and cause back-flooding with large fluctuations of groundwater level, particularly where the recharge variations are high. This is particularly the case if recharge is dominated by allogenic spatially concentrated, but timely highly variable inflow.

**Figure 1:**
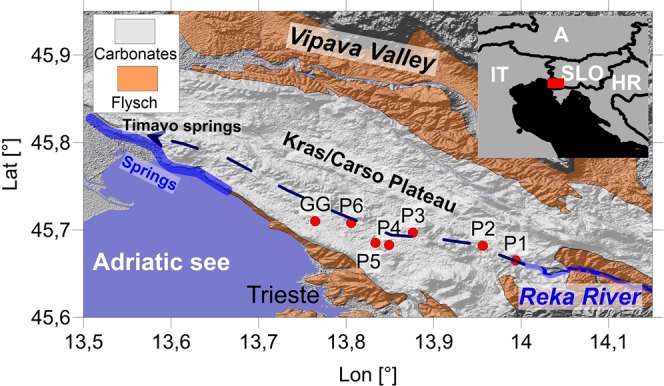
Location map showing the position of the river entering the Karst system underground, and emerging as springs. GG is the Grotta Gigante cave. P1 to P6 are the wells monitoring the underground karstic water system discussed in the text. The small inset map shows the position of the location map (red rectangle); A Austria, IT Italy, SLO Slovenia, HR Croatia. Limit between Carbonate rocks and Flysch after Jurkovšek *et al*.^14^. Relief displays the Shuttle Radar Topography Mission data^36^.

A fine example of such system is the aquifer of the *Kras/Carso Plateau*, which extends between southwest Slovenia and northeast Italy (Fig. 1). The hydrology of the system is highly influenced by the Reka River, which sinks underground at the Škocjan Caves (Škocjanske jame) and emerges ∼40 km northwest on the Adriatic coastline, near Duino, as part of the Timavo Springs. The plateau is made up of a succession of Cretaceous to Lower Paleogene carbonates deposited on the Adriatic–Dinaric Carbonate Platform^13,14^. The carbonates are surrounded by flysch, which provides the input of allogenic water on the SE, while at the same time prevents outflow along the SW boundary. This way, the main flow is forced to follow the Dinaric (SE–NW) direction down to the main Timavo Springs.

**Figure 2:**
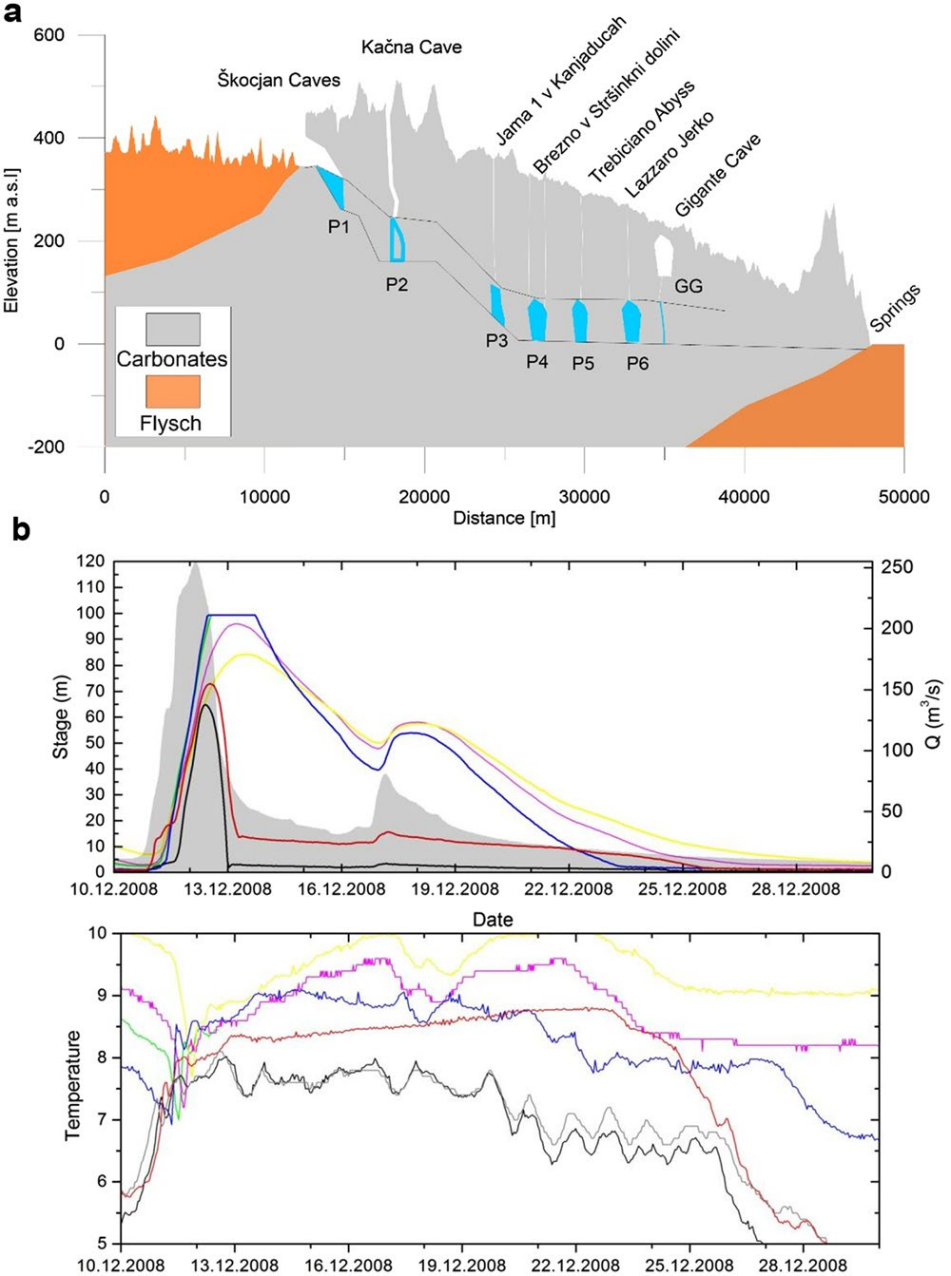
Topographic profile with the karstic water flow system and observation of a pressure pulse along the major Karst conduit for a major flood event. The pulse is contemporaneous over a distance of 20 km and seen in six different caves along the major channel. It lags about 1 day the increase in water flow into the Karst system from the major river (Reka river). The temperature variation in the six caves has a time evolution from one cave to the other, and is delayed with respect to the pressure pulse. (**a**) Topography. In blue and white (only GG) the cross-sections of the caves. Dotted lines give lower and upper boundary of water level in the system. Top level reached during floods. (**b**) Evolution of one flood and level variation in the points P1 to P6. In gray the water flow entering the Karst system. For the same stations P1 to P6 the temperature variation of the water. P1=black; P2 red; P3 blue; P4 green; P5 purple; P6 yellow. Shaded grey area in stage plot and grey curve in temperature plot are relative to Reka.

The system presents a complex speleological environment with many relict caves in the vadose zone, but what is relevant for this work is its epiphreatic zone, a system of conduits and large voids which is affected by large flow fluctuations. An idealized profile showing the position of known caves which reach the epiphreatic zone is shown in Fig. 2a. The caves, marked by P1–P6 were used as monitoring points as decribed below.

Reka River presents the main concentrated allogenic input to the system. Its long term average discharge in the (years 1952–2013) is about 8 m^3^/s^15^. The ratio between the highest and the lowest flow rate is ∼1700, with the maximum measured discharge 305 m^3^/s, and the minimum 0.18 m^3^/s. During flood events the water of the Reka river dominates the Karst system.

The river sinks underground at the Škocjan Caves (P1), continues its way through the Kačna Cave (P2) and then appears in several other caves, which are explored deep enough to reach its flow (P3–P6). The first part of the flow (P1–P3) has a relatively big elevation drop (214 m.a.s.l to about 25 m.a.s.l in 12 km), while in the last 30 km of flow the base level drops by only about 25 m. All caves in the lower part of the system lead to a large chamber at the epiphreatic level.

Due to extreme variability of flow and channel cross-sections, the records of water level in large chambers show oscillations of more than 100 m (see upper dotted line Profile in Fig. 2a), which consequently causes high overpressure in the connecting conduits. These overpressures have been observed and modelled to reach 1 MPa and last for few days to a week.

An example of such flood event from December 2008 is given on Fig. 2b, when the levels between P3–P6, rose between 90 and 130 m. The rate of water level increase was above 5 m/h.

Figure 2a shows the topographic profile with the karstic water flow system. P1 to P6 are caves in which water level is measured continuously. For large flow events the rapid water level increase in the vertical shafts is nearly contemporaneous and increases above 100 m, as seen in Fig. 2b.

**Figure 3:**
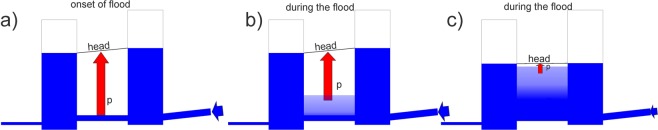
Cartoon showing the pressure increase in the Karst system due to a flood generated by increased water inflow. See text for explanation.

One can consider the system as a series of storage reservoirs connected with conduits. The response of the Reka system has been successfully modeled with such an assumption^16^. The pressure transducers indicate the height of water in the storage reservoirs (in all caves they are placed into large chambers with free water surface). If the velocity head in the conduits is small, most of the head in conduits can be considered as a pressure head. This is particularly true at the onset of a flood (see Fig. 3a). Also in the case that the system is comprised of tight reservoirs and channels, the overpressure in the channel is clearly defined by the head in the adjacent chambers.

During the flood however, if the matrix around the conduit is transmissive, the pressure pushes the water into an adjacent system of smaller fractures and fissures, changing the pressure head distribution. As the matrix is being filled up, the “overpressure” is diminished as shown in the Fig. 3b,c. The overpressure in the conduit therefore diminishes in time.

### Modeling the deformation induced by the pressurized channel

**Figure 4:**
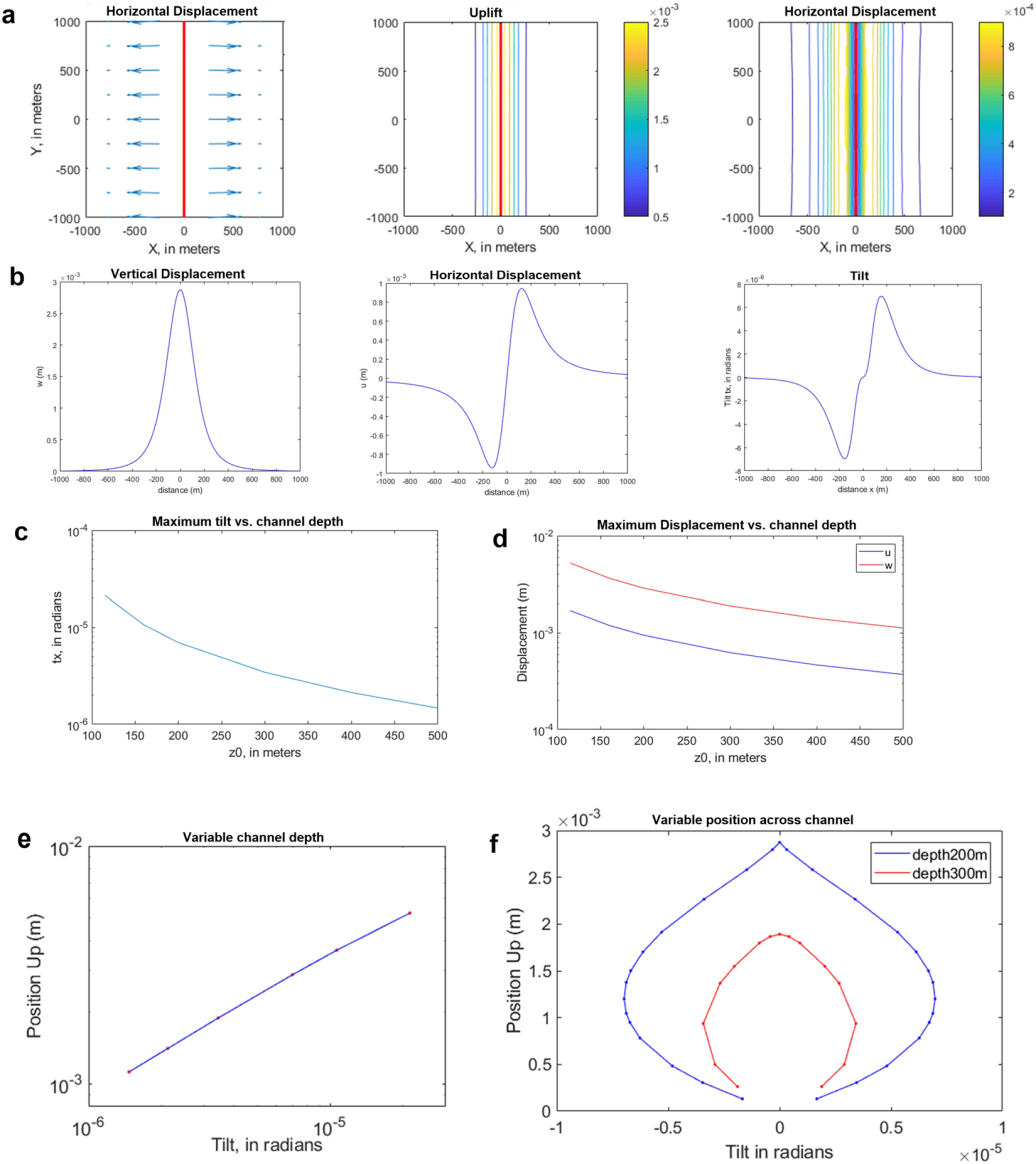
Synthetic model of surface movement for an elongated expanding horizontal channel and parameter exploration with the resulting displacement and tilt. (**a**) Surface dislocation due to the expansion of a horizontal shaft. Center of horizontal channel 200 m deep, elliptical cross-section 60 m wide, 10 m height, to which a pressure of 0.6 MPa is applied simulating a flood event. (**b**) Profile across the channel with vertical and horizontal displacement and tilt. (**c**) Parameter exploration of the channel and the resulting Deformation. Tilt in function of channel depth. (**d**) Displacement in function of channel depth: w = uplift, u = horizontal displacement. (**e**) Maximum uplift versus maximum tilt for varying channel depth. Channel depth varies between 100 m and 500 m. (**f**) Uplift versus tilt for varying position across the channel; channel depth at 200 and 300 m.

Given the hydrologic observations that overpressures up to 1 MPa occur, we investigate whether this signal can be observed by monitoring the ground movement on the surface and below, inside caves. Above the surface we consider the displacements measured with GPS technique, inside caves observations with tiltmeters. We model the pressurized horizontal channel through Finite Element Method reproducing a horizontal channel which is pressurized from its inside, using the actual dimensions of a channel that can be expected in the classical Karst. The predicted surface deformation and tilting are shown in Fig. 4. The movement is an uplift above the channel and an expansion symmetrically on both sides of the channel. The surface movement is reproduced by a channel 4 km long, of elliptical cross-section 60 m wide, and 10 m high, the center at 200 m depth, and dilating due to an internal pressure of 0.6 MPa. Details on the Finite Element Modeling is given in the Methods section. Uplift is in the order of 2.8 mm, horizontal dislocation is 0.9 mm. The amplitude of the displacements is proportional to the applied internal pressure and inversely proportional to the elastic parameters. For modelling purposes standard values for elastic parameters have been used. The tilting follows the direction of the dislocation, with tilting directions orthogonal and away from the shaft, reaching up to 7 µrad. At 500 m distance from the shaft the deformation signals have near to vanished. The decay of amplitude with distance is relatively stronger for tilt, with respect to displacement.

The profile in Fig. 4b traced across the channel, shows that the signal fades away at a few hundred meters from the channel. Relative to the noise level, the signal is easier to be detected in the underground tilt observations, where noise is at the nrad level, than in the GPS records, where noise is at a few mm level.

In order to test the model, the effect of varying depth and length of the channel are explored. Starting from the above parameters, either depth or channel length is changed. The maximum displacement (uplift and horizontal displacement) and tilt are displayed for varying depth of the channel in the graph. The deformation increases by orders of magnitude for a shallower source (Fig. 4c,d). The length of the channel affects the calculated values up to a length of about 1000 m, after which the result hardly changes). This implies that the induced movement is localized over a distance in the order of 1000 m along the channel: if the channel is pressurized over a distance of 1000 m or over a distance of 5000 m, the deformation effect will hardly change. The joint displacement and tilt variation for depths varying between 100 m and 500 m is shown in Fig. 4e, and it is seen that tilt changes over 1.5 orders of magnitude, against displacement that changes over less than one order of magnitude. Tilt is therefore more sensitive to the depth of the channel. Considering the resolution of tilt measurements at the nanorad level, and of GPS at the mm level, it is seen that the induced signal is well above the noise level for tilt, and at the limit of detectability for GPS, at least for the deeper source (smallest values). In Fig. 4f tilt and vertical displacement values are shown for different positions along a NS profile orthogonal to the channel. Moving along the curve of the Up vs tilt graph, the position of the sampled point moves from east to west of the channel. Increasing the distance from the channel, the tilt increases sharply in magnitude, whereas vertical displacement decreases slightly. At greater distances both tilt and displacement decrease. The blue curve is for a shallower channel (200 m depth), red for a deeper channel (300 m depth).

To our knowledge the concept that a sub-horizontal hydrologic karst channel could develop an overpressure that is sufficient to deform and uplift the overlying crust is new. Up to present, the signals have been attributed to the presence of vertical fractures, which would open due to the water infiltration^17–19^.

Given the knowledge on the existing overpressure from the hydrologic models, we find that the uplift and tilting is expected due to the deformation produced by an underground pressurized channel.

### Observed deformation induced by the underground overpressure

The geodetic instrumentation with which we detect a crustal deformation associated with the hydrologic pressure increases, is the GPS receiver and two tiltmeters. The tiltmeters are installed in a cave, which increases the signal-to-noise ratio with respect to an installation on the surface, due to a stable temperature environment, and protection against ambient factors (wind, rain).

We find that during floods the soil is uplifted by a few mm and displaced horizontally and tilted close to the same direction. The movement induced by hydraulic flow is typical to karst systems and the relatively large signal has been noted with GPS, tilt, extensometric and gravity measurements^18,20–22^.

**Figure 5:**
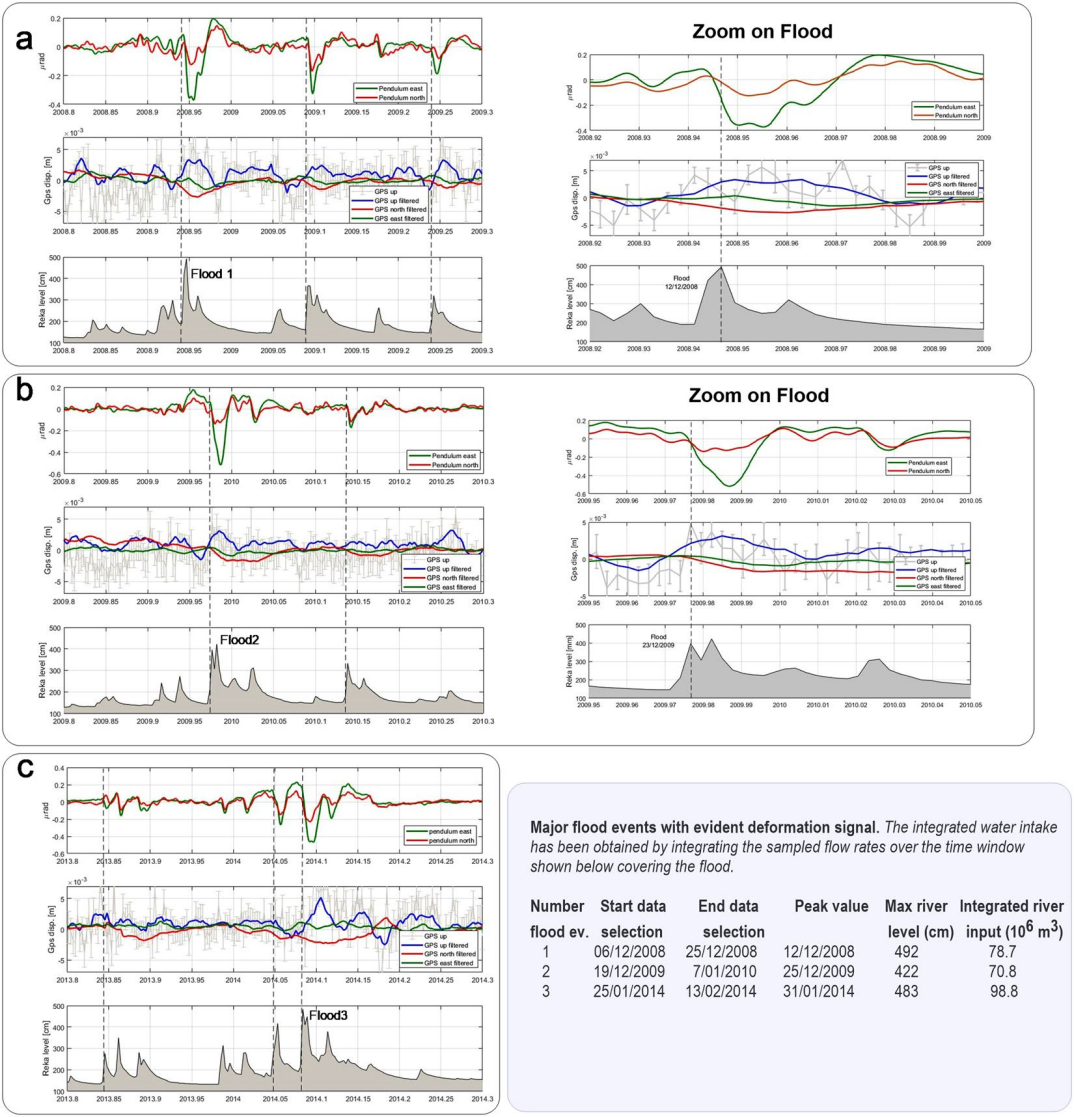
Time series of GPS and tilt, frequency treated to make the hydrologic signal evident. River Reka, GNSS station TRIE low pass filtered at 5 days, horizontal pendulum Grotta Gigante bandpass filtered between 24 and 1440 hours. Time marks of Flood 1, 2, 3 correspond to the strong floods detailed in the Table. Stippled vertical lines mark minor floods. The original pre-filtered GNSS up component is shown with its error bars in grey. (**a**) Time series including flood 1; (**b**) time series including flood 2; (**c**) time series including flood 3.

In Fig. 2 it is seen that the flood event in the river floods the Karst channels, increasing the pressure head, and leading to an increase in water level by over 100 m in different cave shafts. In Fig. 5 the GPS displacement and the tilting deformation for several flood events is shown. The GPS shows an uplift and a movement towards SW, correlated with the hydrologic signal. The tiltmeter also correlates and tilts as well towards SW. The tiltmeter and the GPS receiver are almost at the same location.

The water level increase of the river Reka precedes the tilt and GPS signal by a few days, as it precedes the pressure pulse seen in the piezometers. The tilting is always oriented towards south west with amplitude up to 0.5 microrad, the GPS displacement is an uplift of several millimeters (filtered value up to 3 mm), and a lesser horizontal movement towards south west. The entire deformation movement is recovered after the pressure pulse. The observations find an explanation in a pressure pulse affecting the deep hydraulic channels at a certain distance from the station. The pressure pulse affects the channel over a distance of tens of km, which is demonstrated by the fact that the water level increase is near to contemporaneous in different locations (P1 to P6) along the channel.

The case of the prototype Italian-Slovenian Karst is very well studied and measurements of the deformation and underground water flow exist over many years, which allows us to verify our hypothesis. We think that the phenomenon is common to all karst systems globally. Rocks that are strongly fractured and faulted so that underground water flows collect, are potentially prone to the overpressure, particularly if close to topographic relief in which the water acquires kinetic energy. Some regions have seasonally modulated seismicity, as in the seismic area of northeast Italy^23^. The water flow overpressure could be the explanation of this phenomenon, as it leads to a localized underground stress change due to the pressure of the water acting on the rock. The pressure is comparable to pressures used in geothermal energy exploitation with heat exchange of fluids pressed into the rock or in the case of natural gas temporary storage deposits. Here we demonstrate the existence of the movement and present a model to quantify it. Given our results, the systematic observation of ground movements by a dense network of GNSS stations could be developed into a system for discovery of underground water provisioning channels, in particular in karstic environments. Monitoring of the water pressure in karst channels accessed for recreational purposes could be a further application, and would reduce the hazard of being blocked inside the channels.

## Conclusion

We have shown that in karstic environments during floods the overpressure in the hydrologic conduits is so large, it lifts the surface in the vicinity of the conduit by several mm. The deployment of a great number of GNSS stations forming a two dimensional array could be a new technique to define the underground channels. The location of channels is important for water provisioning, as in karstic environments there is not a diffuse water table, but localized water run offs. This poses great challenges when planning new wells, as along the channels great volumes of water are available, but at less than a meter distance, the vertical drilling would produce a dry well. When choosing the location of an industrial plant with potentially pollutant agents, the opposite will be useful, to select a location far from the water conduits to reduce risk of hydrologic pollution. In both cases the position of the conduits is an essential piece of knowledge. The advent of low cost single-frequency GNSS receivers^24^ prospects a new approach to hydrologic monitoring in karst areas, which are difficult to monitor due to the very localized character of the underground water conduits and absence of water table. Daily repeatability better than 1 mm for baselines up to 3 km and good sky visibility can be achieved^24^. Being the cost of such devices much smaller than the one of classical dual frequency receiver it would be possible to have a set of 10 GNSS stations permanently observing deformation for a period of 1–2 years. After this period the stations can be moved to other points if no signal related to hydrology is observed or the stations can be densified to obtain major insights in the observed deformations. This procedure together with an ad-hoc algorithm to properly recognize the effect in terms of deformation of flood events (maybe based on the correlation between river levels and GNSS observed deformation) could possibly allow to find new channels.

In tectonized areas karstic layers may have been tilted and brought partially to several km depths, maintaining the original karstic fabric with superficial epikarst and large conduits at several 100 m depth from the original surface. These conduits can be still open and allow large fluxes of water to flow rapidly at greater depth, inducing the overpressure at the depths of the possible seismogenic zone, usually above 10 km. This implies that during floods overpressures greater than 1 MPa develop, modifying temporarily the stress field and pressing water into faults. This would be a mechanism to relate stress changes on fault to fluids migration and could be one cause for the seasonal cycles of seismicity observed in different places, as e.g. in North East Italy^23^ and intraplate North America^25^. The deeper the conduits reach, the greater can the overpressure develop, as when they are filled they transfer the pressure of the complete water column, at 1 km depth this corresponds to 10 MPa overpressure. These pressures are one order of magnitude lower than the pressures used typically for temporary gas storage in fault systems, but in the order of stresses (1 MPa) shown to induce seismicity in fluid injection experiments^26^. Induced seismicity by strong rainfall in karstic environments has been reported in Germany^27,28^ and Switzerland^29^. For instance, in the latter case an extreme rainfall had produced peak discharges from a cave of 20 m^3^/sec, which was estimated to be only a portion of the total flux entering the karst system. In this case pressure diffusion from the surface to the fault region at 5 km below the surface was thought to trigger the earthquake on critically stressed faults, and later was proposed to be due to an increase of vertical stress on the underlying poro-elastic medium. The vertical stress was attributed to an increase of the water column above the water table^30^. Coal mines that have been in production for decades constitute a well-drained channel system. Abandoned mines flood with water, if the water is not actively pumped out. The system then has analogies to a karstic flow system, especially when it drains a natural catchment area. Our results suggest that an experiment with monitoring such a system through GNSS and tilt observations could give information on overpressure occurrences.

## Methods

### Hydrologic observations

Programmable automatic pressure transducers and temperature sensors with data loggers were fixed to the level of base-flow in caves marked by P1–P6 on Fig. 2. The logging interval was between 30 and 60 minutes. The measurements took place between 2005 and 2009. Due to the remoteness of observation sites, which were up to 340 m below the ground level, the records are not complete (see Table 1 in Gabrovšek *et al*.^16^) and only few large floods were simultaneously recorded in all caves.

The hydrologic observations of the river Reka which enters the Karst system is available since 1954 with daily sampling.

The river measurements were made until 1956 on regular basis daily (staff-gauge, 1 observation per day, additional observations during high levels). From 1957 to 2006 the gauging station was equipped with a water level recorder (1957–1976 water level recorder “METRA”; 1976–1981 SEBA DELTA; 1981–2006 SEBA –OMEGA with pressure probe) and from 2004 with a data logger. Discharge values are derived from the water level with an empirical transformation function. The function is based on discharge measurements with a current meter and since 2005 with an acoustic Doppler current profiler (ADCP) or flow tracker ultrasonic velocimeter (321 discharge measurements between 1952–2011; precision ±5%; 23 validated rating curves in 37 intervals).

### Details on Finite Element Model

The model considers the effect of the pressurization of the underground water channel on the deformation of the overlying rocks, due to the water flow in the channels. The water level in the shafts up to 40 km distant from the hydrologic intake rises with very small time delay during the floods, and the decay of the high stand starts later than the time delay, also for the most distant stations. The water level rise in the shafts constitutes a pressure gauge, it means that the overpressure is present along the entire discharge conduit. The conduit could be made of one single main arm, or a network of conduits, this is unknown. We use the approximation of an equivalent single linear conduit in the vicinity of the station to model the deformation.

The modeling was done in the COMSOL Multiphysics software environment, in particular using the Structural Mechanics module which is made for structural analysis of general 3D bodies. It is based on the Navier equations for linear stress-strain to estimate displacement, stress and strain of the body. The complete model volume is made up of a parallelepiped (height: 4000 m, width: 8000 m, depth: 4000 m), in which the channel is embedded. The model is discretized with a selective mesh, which is more detailed along the channel and between the channel and the surface, ranging from 0.5 m to 150 m. The material is elastic (Young modulus E = 10^10^ Pa, Poisson ratio ν = 0.25, density ρ = 2400 kg/m^3^) and all boundary faces are free from constraints and loads. The initial values of displacement and velocity are zero, and the boundary load is applied to the internal faces of the channel in the amount of 0.6 MPa, simulating the internal pressure. The fixed constraint, that makes the geometric entity fixed, has been applied to the vertical faces of the outer space that are parallel to the channel, and to the lower horizontal face. The upper surface and the two vertical boundaries parallel to the channel sections are free. We have used the default solver MUMPS (Multifrontal Massively Parallel Sparse Direct Solver). The dimension and depth of the conduit is realistic according to knowledge of the karst hydrologic system.

### Details on geodetic observations

The horizontal pendulums that record the underground water flow are mounted in a natural cave, with bottom at 151 m above sea level, length of 160 m, width of 65 m and height of 107 m. The pendulums are long-base tiltmeters of the horizontal pendulum type with Zöllner suspension^31^ directly mounted between top and bottom of the cave. They consist of a sub-horizontal pendulum arm suspended by an upper wire fixed at the vault of the cave and a lower wire fixed to the ground of the cave. The distance between upper and lower mountings is 95 m and the period of oscillation of the pendulum in the horizontal plane is presently kept at 6 min^31,32^. The pendulums react to horizontal shifts of the upper mounting in respect to the lower mounting (shear) and to rotation of the cave. The beam’s movements are recorded by a laser beam and a photosensitive device with a sampling of 30 values/sec. The static amplification factor for tilt (ratio of the angle of rotation of the beam in the horizontal plane with the tilting-angle of the line connecting upper and lower mountings) is about 24 000. The original optical recording system, on photographic paper, had an amplification of 4.4 nrad/m, since December 2003 the digital system has a resolution of 8 10^−2^ nrad of recorded tilt. In the present study the pendulum data are downscaled to hourly sampling, after applying the anti-aliasing filter. Thanks’ to their dimension (95 m) and to the amount of the data series collected (from 1966 to today) these pendulums are worldwide unique instruments. The hourly data series present a secular trend, a multi decadal oscillation of 31 years, annual and semi-annual variation and the transient hydrologic induced movements, as shown in^31,33^. In order to isolate the hydrologic signal, the time series is band pass filtered with a cosine-tapered frequency filter (corner-periods equal to 240 hours and 30 hours).

The permanent GNSS station (TRIE) is part of the FReDNet geodynamic network (http://www.crs.inogs.it/frednet). The receiver antenna is materialized on the roof of a small building, fixed to a reinforced concrete pillar of the building located just above the horizontal pendulum. The L1 and L2 GPS data have been processed with the Bernese software v.5.0^34^ forming double phase differences of the ionosphere-free linear combinations. We estimate daily station positions in the ITRF08 reference system, modeling the dry and wet troposphere delays, the crustal deformation caused by ocean tidal loading and the GPS antenna phase center position. Further data analysis details are reported in Devoti *et al*.^35^. The daily time series spans a period of 13 years from February 2003 to mid-2016, in which the instrumental offsets, secular trend and seasonal oscillations were filtered out.

We search for the highest water levels in river Reka for the years 2003–2016 in which we have both tilt and GPS observations in Grotta Gigante. These highest levels correspond to floods of the underground river system, observable as rapid rise of the water level in the vertical shafts and rapid increase followed by a slow decay of the water level in the Reka river. We therefore select time windows of 20 days^33^ to describe the entire flood, with 6 days before the river peak, and the remaining days after the peak, during which the flood typically exhausted its effects. In the table of Fig. 5 the time intervals of the major flood events are given, the first two of which are shown in detail in Fig. 5. The extended time series, comprising the two example floods, are also shown. The integrated water intake has been obtained by integrating the sampled flow rates over the indicated time window covering the flood.

The hydrologic tilt signal is the most evident, the GPS signal tends to have a longer time constant in response to the floods. The Up component of TRIE station has greater variability compared to the horizontal components, but systematically after the floods the up component turns positive, and the two horizontal components turn negative. The time marks of the three selected floods are labeled as Flood 1, 2, 3 respectively. The other floods in the time window are marked also with a vertical time mark (dashed vertical line). The guide to identify a hydrologic induced deformation is the tilt signal, which is more evident due to the lower noise level. According to the expectations gained from the modeling, the GPS and tilt signal respond in the order of magnitude of up to a few mm and microradians, respectively, to the floods in the channel. However Fig. 4 shows that the maximum tilt (8 microrad) should be recorded 200 m far from the channel axis, while exactly on the top of the channel the tilt is close to zero. Similar pattern is also observed in the horizontal GPS component, in this case the maximum displacement reaches 1 mm. The vertical displacement on the other hand shows a maximum value of almost 3 mm directly above the channel axis.

From these considerations we see the complementarity of the two deformation measurements and also emerges the potential of the integration of both the techniques in order to estimate the location of the channel.

Figure 5 shows the observed deformation data from the Grotta Gigante during three flood events: the pendulums record maximum tilts of 0.5 microrad which are an order of magnitude lower with respect to the maximum tilt estimated by our model (Tilt in Fig. 4b). This suggest that the tiltmeters should be close to the axis of the channel, or alternatively at distances larger than 600 m. The observed GPS data show systematic uplift up to 2–3 mm and horizontal displacements of about 1 mm. In this case the GPS observation should also be close to the cylinder conduit.

**Figure 6:**
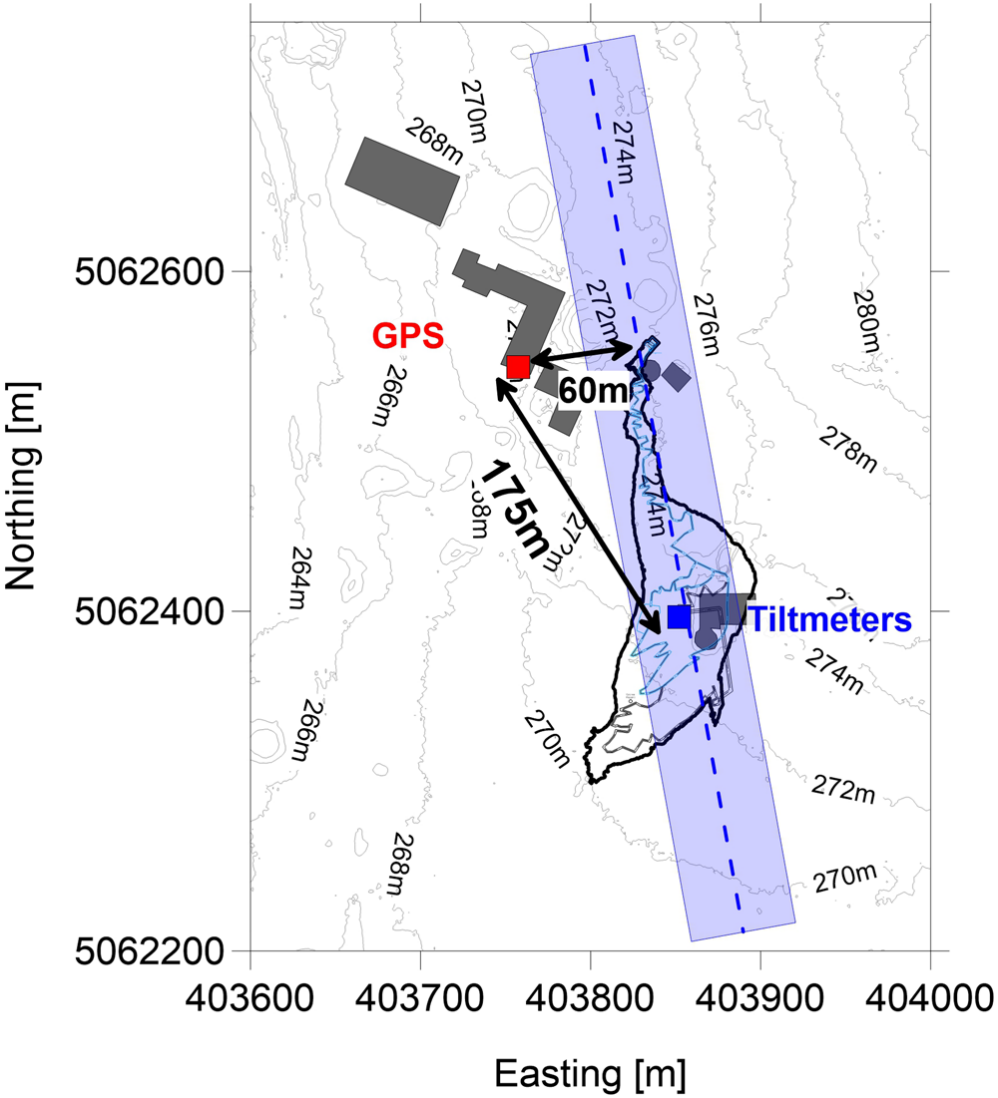
Illustration of the Grotta Gigante area with the location of the geodetic instruments: red square for GPS, blue square for tiltmeters. The Grotta Gigante outline is reported with the thick black line. The presumed location of the channel, based on our model is reported with the light blue area. The distances between the two geodetic instruments and between the GPS and the channel axis are shown with black arrows. Buildings close to the cave marked in gray outline. Topography contour lines after Lidar local DTM^37^.

Figure 6 shows with the red and blue squares the position of the geodetic instruments and one possible location of the channel in blue. The channel should be located with the axis close to the tiltmeters in order to reduce the maximum tilt amplitude, while the GPS station could not be on the axis because a considerable horizontal displacement appears. In the figure the GPS station is about 60 m far from the axis channel, where the model shows also an important uplift up to 3 mm.

### Data deposition

The GPS, tilt, hydrologic data are all publicly available.

## References

[1] Zou R, Freymueller JT, Ding K, Yang S, Wang Q (2014). Evaluating seasonal loading models and their impact on global and regional reference frame alignment. Journal of Geophysical Research: Solid Earth.

[2] Argus DF, Fu Y, Landerer FW (2014). Seasonal variation in total water storage in California inferred from GPS observations of vertical land motion. Geophysical Research Letters.

[3] Amos CB (2014). Uplift and seismicity driven by groundwater depletion in central California. Nature.

[4] Borsa AA, Agnew DC, Cayan DR (2014). Ongoing drought-induced uplift in the western United States. Science.

[5] Barbour AJ, Wyatt FK (2014). Modeling strain and pore pressure associated with fluid extraction: The Pathfinder Ranch experiment. Journal of Geophysical Research: Solid Earth.

[6] Schmidt, D. A. & Bürgmann, R. Time-dependent land uplift and subsidence in the Santa Clara valley, California, from a large interferometric synthetic aperture radar data set. *Journal of Geophysical Research: Solid Earth* **108** (2003).

[7] Xu Z, Bassett SW, Hu B, Dyer SB (2016). Long distance seawater intrusion through a karst conduit network in the Woodville Karst Plain, Florida. Scientific Reports.

[8] Dreybrodt W, Gabrovšek F, Perne M (2005). Condensation Corrosion: A Theoretical Approach. Acta Carsologica.

[9] Ford, D. & Williams, P. *Karst Hydrogeology and Geomorphology*. (John Wiley & Sons,Chichester, England, 2007).

[10] Palmer AN (2007). Variation in Rates of Karst Processes. Acta Carsologica.

[11] Gabrovšek F, Häuselmann P, Audra P (2014). ‘Looping caves’ versus ‘water table caves’: The role of base-level changes and recharge variations in cave development. Geomorphology.

[12] Šebela S (2009). Structural geology of the Škocjan Caves. Acta Carsologica.

[13] Jurkovšek, B., Cvetko Tešović, B. & Kolar-Jurkovšek, T. *Geologija Krasa. Geološki zavod Slovenija*. (Geološki zavod Slovenija, 2013).

[14] Jurkovšek, B. *et al*. Formacijska geološka karta južnega dela Tržaško-komenske planote. Kredne in paleogenske karbonatne kamnine/Geological Map of the Southern part of the Trieste-Komen plateau (Slovenia), 1:50.000 (1996).

[15] ARSO. Arhiv hidroloških podatkov/Archive of hydrological data (2016).

[16] Gabrovšek F, Peric B, Kaufmann G (2018). Hydraulics of epiphreatic flow of a karst aquifer. Journal of Hydrology.

[17] Devoti R, Zuliani D, Braitenberg C, Fabris P, Grillo B (2015). Hydrologically induced slope deformations detected by GPS and clinometric surveys in the Cansiglio Plateau, southern Alps. Earth and Planetary Science Letters.

[18] Grillo B (2018). Cansiglio Karst Plateau: 10 Years of Geodetic–Hydrological Observations in Seismically Active Northeast Italy. Pure Appl. Geophys..

[19] Lesparre N (2017). New insights on fractures deformation from tiltmeter data measured inside the Fontaine de Vaucluse karst system. Geophysical Journal International.

[20] Longuevergne L, Florsch N, Boudin F, Oudin L, Camerlynck C (2009). Tilt and strain deformation induced by hydrologically active natural fractures: application to the tiltmeters installed in Sainte-Croix-aux-Mines observatory (France). Geophysical Journal International.

[21] Jacob, T., Bayer, R., Chery, J. & Moigne, N. L. Time-lapse microgravity surveys reveal water storage heterogeneity of a karst aquifer. *Journal of Geophysical Research: Solid Earth* **115** (2011).

[22] Camp MV (2017). Geophysics From Terrestrial Time-Variable Gravity Measurements. Reviews of Geophysics.

[23] Braitenberg C (2000). Non-random spectral components in the seismicity of NE Italy. Earth and Planetary Science Letters.

[24] Caldera S, Realini E, Barzaghi R, Reguzzoni M, Sansò F (2016). Experimental Study on Low-Cost Satellite-Based Geodetic Monitoring over Short Baselines. Journal of Surveying Engineering.

[25] Craig TJ, Chanard K, Calais E (2017). Hydrologically-driven crustal stresses and seismicity in the New Madrid Seismic Zone. Nature Communications.

[26] Zoback MD, Harjes H-P (1997). Injection-induced earthquakes and crustal stress at 9 km depth at the KTB deep drilling site, Germany. Journal of Geophysical Research: Solid Earth.

[27] Hainzl S, Kraft T, Wassermann J, Igel H, Schmedes E (2006). Evidence for rainfall-triggered earthquake activity. Geophysical Research Letters.

[28] Hainzl S, Ben‐Zion Y, Cattania C, Wassermann J (2013). Testing atmospheric and tidal earthquake triggering at Mt. Hochstaufen, Germany. Journal of Geophysical Research: Solid Earth.

[29] Husen S, Bachmann C, Giardini D (2007). Locally triggered seismicity in the central Swiss Alps following the large rainfall event of August 2005. Geophys J Int.

[30] Miller SA (2008). Note on rain-triggered earthquakes and their dependence on karst geology. Geophys J Int.

[31] Braitenberg C, Romeo G, Taccetti Q, Nagy I (2006). The very-broad-band long-base tiltmeters of Grotta Gigante (Trieste, Italy): Secular term tilting and the great Sumatra-Andaman islands earthquake of December 26, 2004. Journal of Geodynamics.

[32] Braitenberg C (1999). The Friuli (NE-Italy) tilt/strain gauges and short term observations. Annals of Geophysics.

[33] Tenze D, Braitenberg C, Nagy I (2012). Karst Deformations Due To Environmental Factors: evidences from the horizontal pendulums of Grotta Gigante, Italy. Boll. Geofis. Teor. Appl..

[34] Beutler, G. *et al*. Bernese GPS Software Version 5.0 (2007).

[35] Devoti R (2017). A Combined Velocity Field of the Mediterranean Region. Annals of Geophysics.

[36] Jarvis, A., Reuter, H. I., Nelson, A. & Guevara, E. Hole-filled SRTM for the globe Version 4, available from the CGIAR-CSI SRTM 90 m Database (2008).

[37] Friuli Venezia Giulia Region. Local Digital Terrain Model Trieste area. http://irdat.regione.fvg.it/CTRN/ricerca-cartografia/downloadsListAlfa.jsp?tipo=5K&codice=110063&nome=BORGO%20GROTTA%20GIGANTE (2018).

